# Acute myopia and angle closure glaucoma from topiramate in a seven-year-old: a case report and review of the literature

**DOI:** 10.1186/1471-2431-14-96

**Published:** 2014-04-09

**Authors:** Yuna Rapoport, Nancy Benegas, Rachel W Kuchtey, Karen M Joos

**Affiliations:** 1 Vanderbilt Eye Institute, 2311 Pierce Avenue, Nashville, TN 37232, USA

**Keywords:** Acute angle closure, Drug reaction, Glaucoma, Elevated intraocular pressure, Seizures, Acute myopia

## Abstract

**Background:**

A case is reported of acute bilateral myopia and angle closure glaucoma in a 7-year-old patient from topiramate toxicity. This is the second known reported case of topiramate induced acute angle closure glaucoma and third known reported case of topiramate induced acute myopia in a pediatric patient.

**Case presentation:**

This case presents a 7-year-old who had recently begun topiramate therapy for seizures and headache. She developed painless blurred vision and acute bilateral myopia, which progressed to acute bilateral angle closure glaucoma. After a routine eye exam where myopia was diagnosed, the patient presented to the emergency room with symptoms of acute onset blurry vision, tearing, red eyes, swollen eyelids, and photophobia. The symptoms, myopia, and angle closure resolved with topical and oral intraocular pressure lowering medications, topical cyclopentolate, and discontinuation of topiramate.

**Conclusion:**

Acute angle closure glaucoma is a well-known side effect of topiramate, but is rarely seen in children. It cautions providers to the potential ophthalmic side effects of commonly used medications in the pediatric population. It highlights the need to keep a broad differential in mind when encountering sudden onset blurry vision in the primary care clinic, the need for careful consideration of side effects when starting topiramate therapy in a child, and the need for parental counseling of side effects.

## Background

Acute angle closure glaucoma (ACG) from topiramate toxicity is well reported in adults. The largest case series was published in 2004 by Fraunfelder et al. [1] of 83 bilateral and 3 unilateral cases. Of these, almost 50% had been using 50 mg or less of topiramate. Eighty-five percent of these cases occurred within the first 2 weeks, with an overall mean of 7 days. There were 5 cases that occurred within hours when the dose of topiramate was doubled. These reported findings of most likely occurrence within 2 weeks and a dosage under 50 mg have been replicated in another large case series [2].

Topiramate is a sulfamate-substituted monosaccharide and works via blockage of voltage-gated sodium channels, hyperpolarization of potassium currents, enhancement of postsynaptic GABA receptor activity, and suppression of AMPA/kainite receptor. It is absorbed rapidly after oral intake and crosses the blood–brain barrier. It is mostly excreted in the urine, and has an elimination half-life of 21 hours [2]. In children, it was initially approved in July 1999 as adjunctive treatment for patients 2 years of age and older with partial onset seizures. Later, it was approved for seizures associated with Lennox-Gastaut syndrome, generalized tonic clonic seizures, and as initial monotherapy for partial onset or primary generalized epilepsy. Topiramate has been approved in the adult population as preventive therapy for headache and migraine and is used off-label for these conditions in the pediatric population. In 2011, the pediatric population (0–16 years) accounted for 7% of total use of topiramate with 2.1 million prescriptions and 315,000 patients; 81% of pediatric patients were aged 10–18 years [3].

Acute myopia and angle closure glaucoma are two of many adverse side effects of topiramate. The underlying mechanism of acute myopia and acute angle closure glaucoma is a ciliochoroidal effusion. This leads to ciliary body edema which causes relaxation of zonular fibers, lens thickening, and anterior displacement of the lens -iris complex. The iris bowing forward physically blocks the drain of the eye, preventing aqueous fluid drainage. This ultimately causes secondary ACG and myopia. The ciliochoroidal effusion caused by sulphonamides is an idiosyncratic response in the uveal tissue, and is dose independent [4]. The hapten hypothesis postulates that reactive drug metabolites bind to proteins, forming altered proteins, which are recognized as foreign substances and incite immune reactions [4]. A patient must receive a sensitizing dose prior to inciting the immune reaction with the subsequent dose. The risk of any adverse reaction to a sulfonamide is 3% [5].

Most common ocular signs of acute ACG from topiramate include abnormal vision, acute intraocular pressure elevation, acute myopia [6], microcystic corneal edema, shallow anterior chamber [1], circumciliary congestion, retinal striae [7], macular folds, choroidal detachments, and ciliochroidal detachments [8]. Besides topiramate, other sulfonamides have been reported to cause a similar clinical syndrome, including acetazolamide [9], sulfasalazine [10], hydrochlorothiazide [10], and indapamide [4,11]. All ocular findings are reversible if recognized early and the drug is discontinued.

Treatment includes immediate discontinuation of topiramate, aqueous suppressants including oral or intravenous (IV) acetazolamide and IV mannitol, topical beta blockers, topical carbonic anhydrase inhibitors, topical prostaglandin analogues, and topical cycloplegics such as cyclopentolate or atropine, which work by relaxing the ciliary processes and deepening the anterior chamber. Acute angle closure usually resolves within 24–48 hours with medical treatment, and myopia resolves within 1–2 weeks of discontinuing the topiramate. If refractory, other measures reported to be successful include oral/ IV steroids [12], argon laser peripheral iridoplasty [13], and surgical intervention including choroidal drainage [14], vitrectomy, cataract extraction/ intraocular lens placement, and other glaucoma surgeries.

While there are numerous case reports in the literature of adults presenting with acute ACG from topiramate toxicity in addition to the two case series mentioned above, there are very few case reports in children. One article reports acute myopia in an 8-year-old male with a 6 diopter myopic shift without ACG [15], and one reports a 5-year-old female presenting with acute ACG and acute myopia [16].

This is the second known reported case of acute ACG in a pediatric patient. In this case report, we discuss the presentation, treatment, and resolution of symptoms, and discuss a differential diagnosis of pediatric narrow angles and of elevated intraocular pressure in a child with seizures. We discuss the potential differences between presentation and treatment in the adult and pediatric population. We stress the importance of careful consideration of side effects when starting topiramate therapy in a child, and the need for parental counseling of side effects.

## Case presentation

### History of present illness and review of systems

A 7-year-old female with a history of seizures and headaches presented to the pediatric emergency room (ER) with acute onset of blurry vision. The morning of presentation, she awoke with blurry vision. She presented to her pediatrician, who referred her to an optometrist, where she was refracted to a visual acuity (VA) of 20/20 with a myopic refraction. Visual acuity had been 20/20 at distance previously. Acute myopia is not an altogether unusual presentation to an optometrist and generally a new myopia patient does not cause alarm. She continued to experience worsening vision accompanied with red eyes, swollen eyelids, excessive tearing and photophobia. She denied pain, burning, itching, mucus discharge, or pain with extraocular movements. She denied systemic symptoms including dizziness, nausea, vomiting, malaise, neck stiffness, fever, or any focal neurological complaints. Her last dose of topiramate was 25 mg, 20 hours prior to presentation.

### Past medical, surgical, ocular, medication, social and allergic history

She had had 3 focal seizures from age 3–4, and had been treated with levetiracetam until 3 months prior to presentation, having been seizure free for three years. Her headaches returned, and she had one seizure while in school, which consisted of dysconjugate eye movements and brief unresponsiveness. After consultation with her primary pediatrician and her neurologist, topiramate 25 mg at bedtime was started 2 weeks prior to presentation. Her only other medication was cyproheptadine 5 ml at bedtime, as needed. She had no other medical or surgical history. Social, allergic and family history was noncontributory. She was developing normally.

### Examination

In the emergency room, her VA without correction was light perception at 20 feet in both eyes, count fingers at 14 inches in both eyes, and 20/20 at 3 inches in both eyes. In a complete ophthalmologic examination, vision is typically tested at distance (20 feet) and at near (14 inches). In this patient’s case, vision was also checked at 2 inches because anteriorization of the lens leading to acute myopia was suspected. Pupils were 6 mm, minimally reactive, with no relative afferent papillary defect in either eye. Extraocular movements were intact. Fields were constricted 360 degrees to confrontation in both eyes. Intraocular pressure (IOP) measured 40 mmHg in the right eye and 41 mmHg in the left eye, as measured by tonometry using a portable tonopen. External exam was significant for slightly edematous upper and lower lids. On slit lamp exam, sclera and conjunctiva showed 1+ injection, with conjunctival chemosis temporally bilaterally. There was no corneal edema. The anterior chamber was diffusely shallow bilaterally. The irides were round with a regular insertion, without iris bombe. There was irido-corneal touch as seen by the Van Herick method [17]. The lenses were clear. Fundus exam showed a pink and healthy optic disc in both eyes with sharp margins, with a cup: disc ratio of 0.25. The rest of the fundus examination was unremarkable. No choroidal effusions were seen.

### Treatment

Upon diagnosis with topiramate-induced acute ACG, she was treated with oral acetazolamide 10 mg/kg × 2 doses, and 5 rounds of topical dorzolamide/timolol and topical bimatoprost in both eyes. Her IOP decreased to 28 mmHg in the right eye, 29 mmHg in the left eye, and her symptoms improved. She was no longer photophobic, the eyelid edema had subsided, and VA was now 20/60 at 14 inches. She was discharged home overnight with topical dorzolamide/timolol twice daily in both eyes, oral acetazolamide 10 mg/kg three times daily × 3 days, and topical cyclopentolate three times daily in the right eye. Topiramate was discontinued and was placed on her allergy list.

### Clinical course

**Figure 1:**
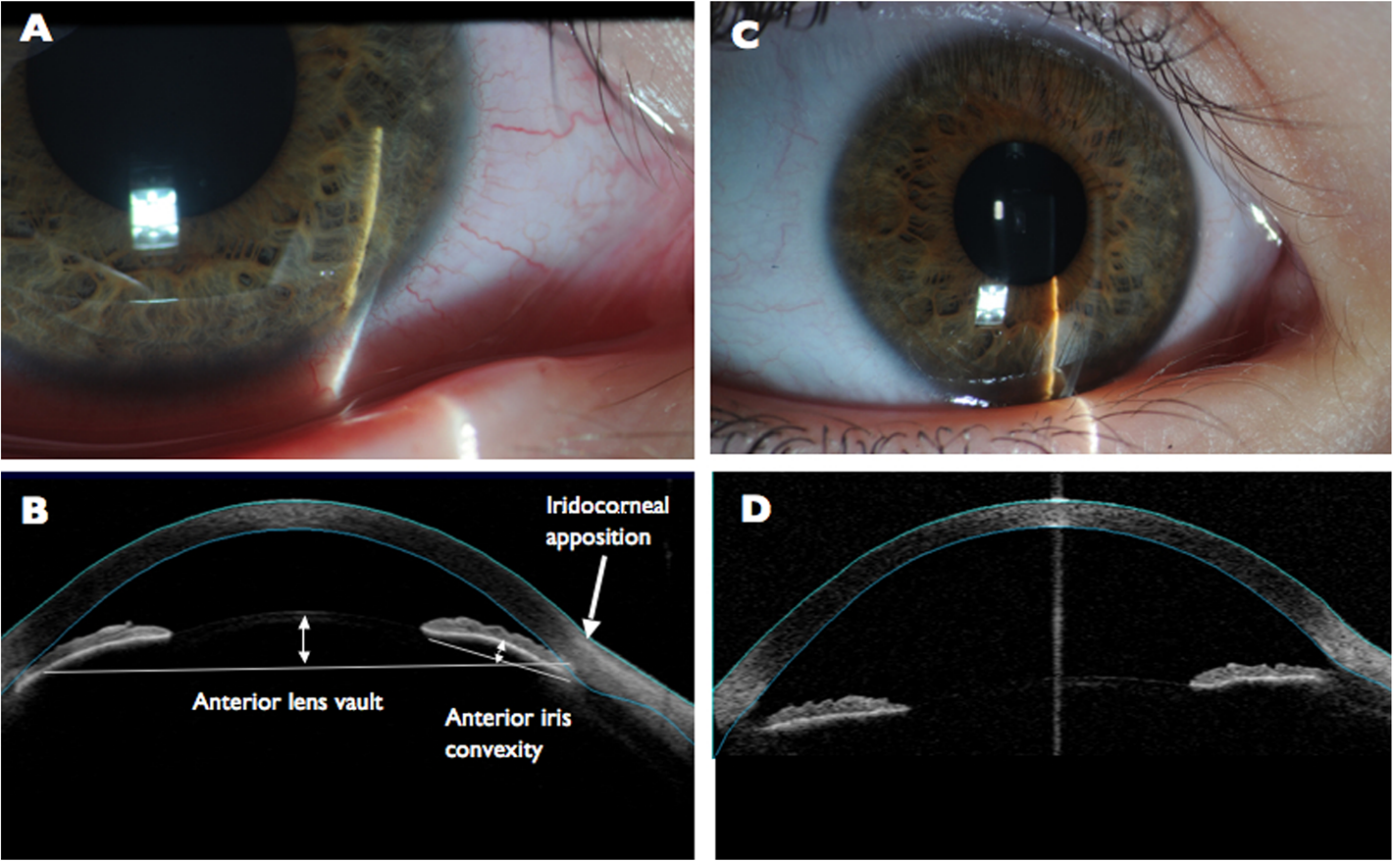
**Clinical and imaging findings. A.** Slit lamp exam of the right eye demonstrating diffusely shallow AC, large pupils, no K edema, and slightly injected conjunctiva. **B.** Normal slit lamp photograph of the right eye after resolution of acute angle closure **C.** Anterior segment OCT of the right eye demonstrating abnormal anterior iris convexity, iridocorneal apposition at the angle, and an anterior lens vault **D.** Normal anterior segment OCT of the right eye demonstrating horizontal iris, no iridocorneal apposition, anterior iris convexity, or anterior lens vault.

She was followed in the clinic closely. The day after her initial presentation, her VA was 20/200 in the right eye, 20/150 in the left eye at distance (20 feet), and 20/60 in the right eye, 20/40 + 3 in the left eye at near (14 inches); IOP was 23 mmHg in the right eye, 21 mmHg in the left eye, and all medications were continued. Her slit lamp examination was unchanged (Figure 1a). In the pediatric population in whom gonioscopy examination is very difficult, anterior segment optical coherence tomography (OCT) is a useful way of discerning anatomy of the angle and the anterior chamber. Anterior segment OCT (Visante; Jena, Germany) in our patient showed anterior iris convexity, iridocorneal apposition at the angle, and an anterior lens vault in both eyes (Figure 1c). All medications were discontinued at eight days after presentation, when her VA was 20/25 + 3, 20/25 at distance, and IOPs were 12 mmHg in the right eye, 11 mmHg in the left eye. At her last follow up visit 2 months after initial presentation, VA and IOP were normal off medications. She had remained headache and seizure free. Repeat slit lamp photograph (Figure 1b) and anterior segment OCT (Figure 1d) demonstrated return to normal anatomy. The anterior chambers were deep, and there was no iridocorneal touch, anterior iris convexity, or anterior lens vault in both eyes.

## Conclusion

This case highlights the fact that drug-induced angle closure, while rare in the pediatric population, should be suspected in children who present with acute onset of blurry vision and have features of bilateral acute angle closure, myopic shift and elevated intraocular pressure. Other considerations in a child with narrow angles similarly caused by a ciliochoroidal effusion include scleritis, uveitis, juvenile idiopathic arthritis, tumors such as retinoblastoma and medulloepithelioma, and exudative retinal detachments. A full differential is presented here, organized by mechanism (Table 1). The patient’s history of seizures and presentation with elevated IOP is in hindsight clearly linked to topiramate, but upon initial consideration those two features could be linked in and of themselves. Childhood phacomatoses such as Sturge Weber, Klippel-Trenaunay-Weber Syndrome, Wyburn-Mason syndrome, tuberous sclerosis, and neurofibromatosis I, as well as Aicardi Syndrome, the Ring 14 Syndrome [18], and CASK mutation [19] all present with epilepsy in childhood and have different mechanisms of elevating IOP (Table 2).

**Table 1:** Differential diagnosis of childhood narrow angles

**Anterior segment dysgenesis**	**Posterior ‘Pushing’ mechanism**
Corneal anomalies: microcornea, cornea plana/sclerocornea	**Anterior rotation of ciliary body**
Axenfeld-Rieger syndrome, Peters anomaly, Aniridia	Nanophthalmos, Inflammation (scleritis, uveitis, juvenile idiopathic arthritis)
Ectopia lentis (trauma, homocystinuria, Marfan syndrome, Ehlers’-Danlos, syndrome, Weill-Marchesani syndrome	Drug-induced
**Pupillary block**	**Pressure from posterior segment**
Aphakia	Tumor (retinoblastoma, medulloepithelioma)
Microspherophakia	Exudative retinal detachment
**Anterior “Pulling’ mechanism without pupillary block**	**Contraction of retrolental tissue**
Neovascular (tuberous sclerosis)	Persistent fetal vasculature
Peripheral anterior synechiae	Retinopathy of prematurity

**Table 2:** Differential diagnosis of elevated intraocular pressure and seizures in children

**Aicardi syndrome**	**Microphthalmos**	**Agenesis of corpus callosum, depigmented chorioretinal lacunae**
CASK mutation	Anterior segement dysgenesis, megalocornea	Dystonia, psychomotor retardation, severe, intellectual disability, scoliosis, mild, dysmorphism, progressive microcephaly
Klippel-Trenaunay-Weber syndrome	Increased episcleral venous pressure	Port-wine stains, venous and lymphatic malformations, soft tissue hypertrophy of affected limbs
Neurofibromatosis	Anterior segment dysgenesis	Optic nerve glioma, Lisch nodules, café au lait spots, neurofibromas, freckling of intertriginous areas
Ring 14 syndrome	Unknown	Macular white spots, strabismus, short, stature, microcephaly, scoliosis
Sturge Weber syndrome	Increased episcleral venous pressure	Port-wine stains, ipsilateral leptomeningeal, vascular malformations
Tuberous sclerosis	Anterior segment neovascularization, retinal detachment	Retinal astrocytic hamartomas, ash-leaf spots, adenoma sebaceum, cardiac rhabdomyoma
Wyburn-Mason syndrome	Intraocular hemorrhage	Retinal racemose hemangiomas, arteriovascular, malformation with dilated and tortuous shunt vessels

Diagnosis Intraocular Pressure Elevation Mechanism Other Clinical Findings.

A thorough history and examination should eliminate most of the previous entities from consideration. As this is the second known reported independent case report of childhood ACG from topiramate, it is difficult to draw conclusions as to differences between the clinical presentation and course between children and adults. Since they are both caused by the same mechanism, the same course and outcome is assumed to occur. Treatment is the same as in adults except topical alpha agonists are contraindicated in children. Further investigation and reports will help elucidate potential differences between adults and children.

In comparison with the one other case of pediatric ACG from topiramate [16], our patient did not present with headache, nausea, and fatigue, had a slightly lower IOP (40 vs. 50 mmHg), did not show microcystic corneal edema, but did have iridocornreal touch. In addition to pressure-lowering medications, a thorough review of systemic medications should be undertaken with discontinuation of the most probable causative agent. A high index of suspicion for drug-induced causes will allow for a quick diagnosis and complete visual recovery.

Important clinical pearls of topiramate toxicity induced angle closure glaucoma were highlighted by this case report. The first presenting sign of acute angle closure from topiramate toxicity may be blurring of vision bilaterally at distance with normal vision at near, representing the myopic shift, and occurs prior to symptoms and elevated IOP. Eighty five percent of cases of IOP elevation occur within two weeks of use. Finally, primary narrow angle glaucoma is rare under 40 years of age, and secondary angle closure glaucoma, particularly drug-induced ACG, must be considered in pediatric patients.

### Requesting consent statement

Written informed consent was obtained from the patient’s parent for publication of this case report and any accompanying images. A copy of the written consent is available for review by the Editor of this journal.

## Abbreviations

ACG: Angle closure glaucoma; EEG: Electroencephalogram; ER: Emergency room; IOP: Intraocular pressure; IV: Intravenous; OCT: Optical coherence tomography; VA: Visual acuity.

## Competing interests

The authors declare that they have no competing interests.

## Authors’ contributions

YR was involved in the patient care, performed the literature review, and drafted the manuscript. NB was involved in the patient care and edited the manuscript. RK was involved in the patient care and edited the manuscript. KJ analyzed the data and was involved in the manuscript drafting and critical revision for content. All parties approved the final manuscript.

## Pre-publication history

The pre-publication history for this paper can be accessed here:

http://www.biomedcentral.com/1471-2431/14/96/prepub
